# Swine Influenza in Sri Lanka

**DOI:** 10.3201/eid1903.120945

**Published:** 2013-03

**Authors:** Harsha K. K. Perera, Geethani Wickramasinghe, Chung L. Cheung, Hiroshi Nishiura, David K. Smith, Leo L. M. Poon, Aluthgama K. C. Perera, Siu K. Ma, Narapiti P. Sunil-Chandra, Yi Guan, Joseph S. M. Peiris

**Affiliations:** Author affiliations: University of Hong Kong, Hong Kong Special Administrative Region, People’s Republic of China (H.K.K. Perera, C.L. Cheung, H. Nishiura, D.K. Smith, L.L.M. Poon, S.K. Ma, Y. Guan, J.S.M. Peiris);; University of Kelaniya, Kelaniya, Sri Lanka (H.K.K. Perera, N.P. Sunil-Chandra); Medical Research Institute, Colombo, Sri Lanka (G. Wickramasinghe);; Japan Science and Technology Agency, Kawaguchi, Japan (H. Nishiura);; Colombo Municipal Council, Colombo (A.K.C. Perera)

**Keywords:** swine influenza, Sri Lanka, epidemiology, viruses, spillover, ecology, A(H1N1)pdm09, influenza

## Abstract

To study influenza viruses in pigs in Sri Lanka, we examined samples from pigs at slaughterhouses. Influenza (H3N2) and A(H1N1)pdm09 viruses were prevalent during 2004–2005 and 2009–2012, respectively. Genetic and epidemiologic analyses of human and swine influenza viruses indicated 2 events of A(H1N1)pdm09 virus spillover from humans to pigs.

Data on swine influenza in southern Asia are limited (*1**–**3*). Sri Lanka is an island in this region with a human population of 21 million and a swine population of ≈83,785 (*4**,**5*). Pigs are not routinely imported into Sri Lanka. Most (61%) swine farms are located in the western costal belt spanning the Puttlam, Gampaha, Colombo, and Kalutara districts. In 2010, for these 4 districts, pig population densities were 7, 15, 12, and 1 animal per km^2^, respectively (*4**,**5*). In 2001, for these districts, the human population densities were 246, 1,539, 3,330, and 677 persons per km^2^, respectively (*6*).

## The Study

During 2004–2005 and 2009–2012, tracheal and nasal swab and serum samples were collected from pigs at government slaughterhouses in Sri Lanka (Table 1). Culture tubes with MDCK cells were inoculated with the swab samples, and 2 blind passages were made. Also, embryonated eggs were inoculated by the allantoic route with swab samples collected during 2004–2005. Virus isolates were subtyped by hemagglutination inhibition (HAI) testing and neuraminidase inhibition testing with reference antiserum as described (*7**,**8*), and results were confirmed by sequencing the hemagglutinin and neuraminidase gene segments.

**Table 1:** Swine influenza viruses isolated from pigs, Sri Lanka*

Collection years, location	No. pigs sampled/no. viruses isolated (source)
2004–2005	
Welisara†	40/0
Dematagoda‡	260/1 (tracheal swab)
2009–2012, Dematagoda‡	2,710/26 (7 tracheal swabs, 19 nasal swabs)

*From each pig, 1 tracheal swab, 1 nasal swab, and 1 serum sample was collected, except during 2009–2012, when only 1,773 serum samples were collected from the 2,710 pigs beginning in February 2010. †National Livestock Development Board swine farm, Welisara, Sri Lanka (slaughters 2–3 pigs/ wk). ‡Government slaughterhouse, Colombo Municipal Council, Dematagoda, Colombo, Sri Lanka (slaughters 10–20 pigs/d).

RNA extraction, cDNA synthesis, PCR, genome sequencing (*9*), and one-step quantitative real-time reverse transcription (RT-PCR) for rapid genotyping of all 8 gene segments (*10*) of A(H1N1)pdm09 isolates were performed as described. Methods used for the phylogenetic analysis are described in Technical Appendix 1. GenBank accession numbers assigned to the sequences determined in this study are KC197816–KC197855 and KC190041–KC190078.

Serum samples were tested by HAI as described (*8*) by using the virus antigens shown in Table 2. The number of human influenza A(H1N1)pdm09 viruses detected by RT-PCR in Sri Lanka during July 2009–March 2012 was obtained from the World Health Organization FluNet and from the Epidemiology Unit, Ministry of Health, Sri Lanka (*11**,**12*). Seven A(H1N1)pdm09 viruses isolated from humans during 2009–2011 were obtained from the National Center for Influenza, Medical Research Institute, Sri Lanka, and were genetically sequenced as described above. Genetic sequence data of the hemagglutinin gene of 5 additional human A(H1N1)pdm09 viruses isolated in Sri Lanka were provided by the World Health Organization Influenza Collaborating Centre, Melbourne, Australia.

**Table 2:** Homologous serological reaction profile to subtypes of influenza viruses among pigs, Sri Lanka, 2004–2005 and 2010–2012*

Virus antigen (lineage)	Seroprevalence, no. (%)				
	Jan 2004–Mar 2005, n = 300	Feb–Aug 2010, n = 149	Sep 2010–Mar 2011, n = 284	Apr–Oct 2011, n = 577	Nov 2011–May 2012, n = 763
A/swine/Colombo/48/2004 (H3N2) (human-like)	185 (61.6%)	06 (4.0%)	0	0	0
A/swine/HK/2422/98 (H3N2) (swine) (human)	0	0	0	0	0
A/Sydney/5/97 (H3N2) (human)	0	0	0	0	0
A/swine/HK/1774/99 (H3N2) (European swinelike)	0	0	0	0	0
A/HK/44062/2011 (H3N2) (human)	Not tested	Not tested	0	0	0
A/swine/Colombo/330/2009 (H1N1) (H1N1pdm09)	0	16 (10.7)	95 (33.5)	14 (25.1)	77 (10.1)
A/swine/HK/29/2009 (H1N1) (Eurasian avian)	0	01 (0.6)	0	0	0
A/swine/HK/1110/2006 (H1N1) (North American–triple reassortant)	0	01 (0.6)	0	0	0
A/swine/HK/915/2004 (H1N2) (North American–TR)	0	0	0	0	0
A/swine/HK/4167/99 (H1N1) (classical swine)	0	0	0	0	0
A/swine/Ghent/G112/2007 (Eurasian avian)	0	0	0	0	0

*Hemagglutination inhibition reciprocal antibody titers ≥40 were considered positive. The range of the antibody titers was 40 to ≥1,280. If serum reacted to multiple antigenically related influenza H3 or H1 subtype viruses, we categorized the serum as having a homologous reaction profile to the virus to which titer was ≥4-fold higher than that for other viruses of the same subtype. For example, during 2004–2005, some serum samples were seropositive for influenza A/swine/HK/2422/98 (H3N2) virus; however, because in the same sample, titer to influenza A/swine/Colombo/48/2004 (H3N2) virus was >4-fold higher, reactivity was attributed to the latter.

One influenza A virus, A/swine/Colombo/48/2004(H3N2), was isolated in MDCK cells from a tracheal swab sample collected in 2004–2005. All genes of this virus were closely related to human influenza (H3N2) virus isolate A/Ragama/190/2003 from Sri Lanka and to other subtype H3N2 influenza viruses isolated worldwide at this time (data not shown). During January 2004–March 2005, a total of 185 (61.6%) of 300 serum samples tested were positive for A/swine/Colombo/48/2004(H3N2); HAI titers ranged from 40 to >1,280 (Table 2), indicating that this human-like influenza (H3N2) virus was widespread in the swine population. Serum samples collected from swine during 2009–2012 were also mostly seronegative to this and to more contemporary human influenza (H3N2) viruses.

**Figure 1:**
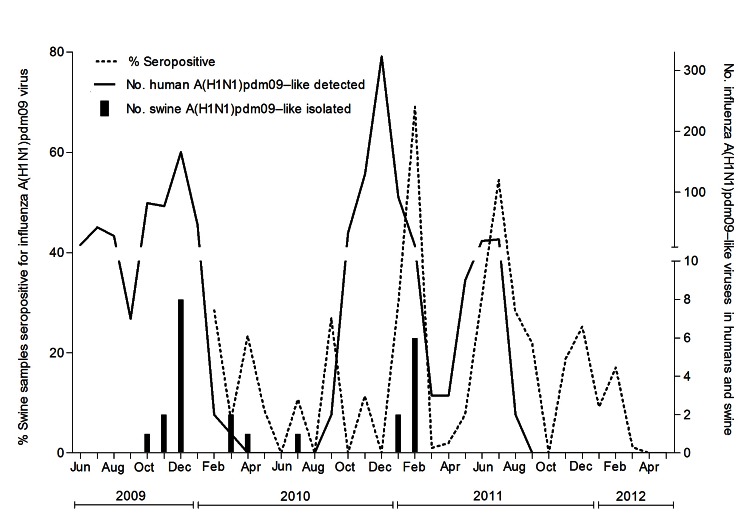
Distribution of percentage of swine serum samples seropositive for influenza A(H1N1)pdm09 viruses, by month, and number of A(H1N1)pdm09 viruses detected in humans and swine. The left y-axis represents the percentage of swine serum samples positive for A(H1N1)pdm09 virus. The right y-axis represents the number of swine A(H1N1)pdm09 isolated in the study and reverse transcription PCR–positive human A(H1N1)pdm09 detected in Sri Lanka.

Of the nasal and tracheal swab samples collected from 2,710 pigs during 2009–2012, a total of 26 (0.5%) viruses were isolated in MDCK cells; all were identified as A(H1N1)pdm09 viruses. All 8 gene segments of these viruses were similar to those of A(H1N1)pdm09 virus; no evidence of reassortment with other swine or human viruses was found. These isolates were collected on 12 sampling occasions from apparently healthy pigs on 7 farms. The 2 peaks of A(H1N1)pdm09 detection in swine followed peaks of human A(H1N1)pdm09 outbreaks that occurred during June 2009–January 2010 and October 2010–February 2011, which represented the first and second waves of the pandemic in Sri Lanka (Figure 1). Overall, virus yield was higher from nasal swab samples than from tracheal swab samples (Table 1).

**Figure 2:**
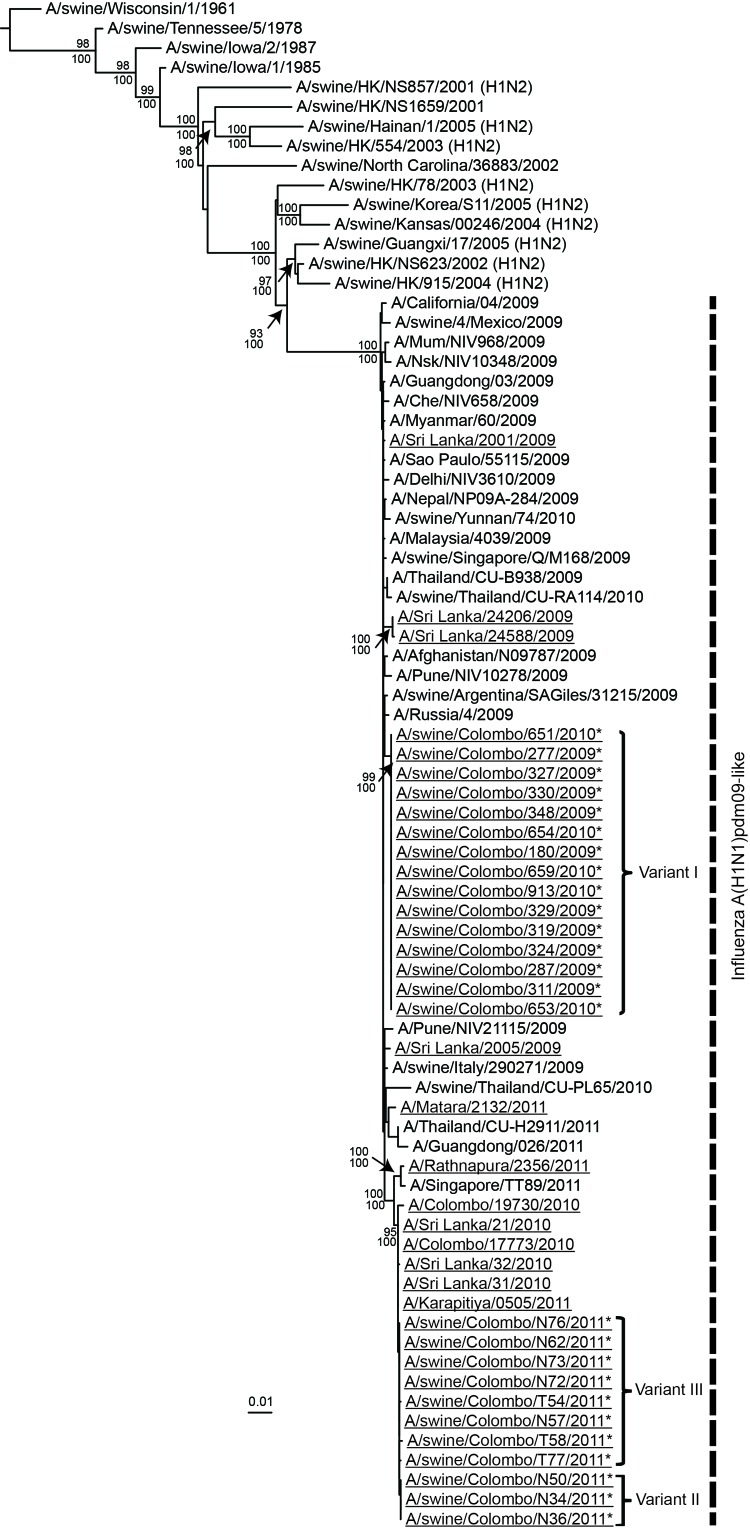
Phylogenetic relationship of the hemagglutinin 1 gene of the human and swine influenza A(H1N1)pdm09–like viruses isolated during 2009–2012 in Sri Lanka. Underlining indicates swine and human viruses characterized in this study; *indicates swine A(H1N1)pdm09 virus isolates. Nucleotide sequences from selected, related avian, equine, swine, and human virus strains available in GenBank are included for comparison. The phylogenetic tree was generated by the maximum-likelihood method and rooted to A/duck/Miyagi/66/77(H1N1) virus (Technical Appendix 1). Scale bar represents number of nucleotide substitutions per site. Vertical dashed line indicates influenza A(H1N1)pdm09–like virus lineage. Branch labels record the stability of the branches >500 bootstrap replicates. Numbers above and below branches indicate neighbor-joining bootstrap values and Bayesian posterior probabilities, respectively. Only bootstrap values ≥70% and Bayesian posterior probabilities ≥95% are shown. Three genetic variants with ≥1 aa difference in hemagglutinin 1 are indicated.

Phylogenetic analysis showed that the 15 A(H1N1)pdm09 viruses isolated from swine during October 2009–July 2010 clustered together and with other A(H1N1)pdm09 viruses isolated from humans during this period. In contrast, swine A(H1N1)pdm09 viruses isolated in 2011 clustered separately from swine viruses isolated during 2009−2010 and clustered with human A(H1N1)pdm09 viruses isolated in 2010 and 2011 in Sri Lanka and elsewhere (Figure 2). The amino acid signature changes occurring within human A(H1N1)pdm09 viruses in the first and second pandemic waves are reflected in the corresponding waves of swine infections, and each linage that occurred in swine led to extinction (Technical Appendix 2). This finding suggests that the A(H1N1)pdm09 infections among swine that occurred during January–February 2011 were separate spillover events from the second wave of human infections during October 2010–February 2011 rather than from continued epizootic transmission among swine from October 2009. The upper 95% CI of the prevalence of each viral genetic variant, given no positive isolates since the last detection, declined to almost zero during the course of observation, indicating probable extinction of these genetic variants (Technical Appendix 2).

On some sampling occasions, A(H1N1)pdm09 viruses were isolated from multiple pigs from the same farm, and on 1 sampling occasion, isolates came from multiple pigs (from the same farm) slaughtered 9 days apart. In such instances, with 1 exception, we found viruses from the same farm to be genetically identical, suggesting continued circulation of the virus in swine herds.

Swine serum samples collected in 2004–2005 showed no seroprevalence to A(H1N1)pdm09, A/California/4/2009, and A/swine/Colombo/330/2009 viruses (Table 2). In 2010, seroprevalence to A(H1N1)pdm09 virus was detected (Figure 1). After peaking in February 2011, seroprevalence declined to undetectable levels in April–May 2012, suggesting that the A(H1N1)pdm09 virus was not sustaining transmission among pigs in the absence of continued human infection. The maximum cross-correlation between incidence of human and swine virus isolates was found after an 8-week lag, indicating that the rise in incidence of human virus preceded that in swine by 7–8 weeks (Technical Appendix 2).

## Conclusions

Isolation of human-like influenza A (H3N2) and A(H1N1)pdm09-like viruses from pigs in Sri Lanka probably represents spillover infection from humans, with self-limited transmission and extinction within pig herds. This finding might reflect characteristics of swine husbandry in Sri Lanka, where swine population density in the study area is relatively low (7.7 pigs/km^2^), or other factors (*5**,**13*). Genetic characterization of individual gene segments of all influenza (H3N2) and A(H1N1)pdm09 viruses from swine showed no evidence of genetic reassortment. This finding contrasts with those from Hong Kong, Thailand, Argentina, and the United States, where reassortment of A(H1N1)pdm09 with other swine influenza viruses has reportedly occurred (*14**,**15*). This contrast might reflect the low prevalence of other swine influenza virus lineages (e.g., classical swine, Eurasian avian-like and triple-reassortant swine) endemic to Sri Lanka. With the exception of subtype H3N2 viruses (Table 2), no evidence of other endemic swine influenza viruses circulating in swine in the country before the emergence of the A(H1N1)pdm09 in 2009 was found, and influenza (H3N2) virus in swine became extinct around the time of the spillover of A(H1N1)pdm09 to swine. These observations might explain the lack of emergence of A(H1N1)pdm09 reassortants among swine. It might also indicate that A(H1N1)pdm09, although able to spill over from humans to swine, is not ideally adapted to establish sustained transmission among swine in the absence of further reassortment with other swine influenza virus lineages.

Technical Appendix 1 Methods used in phylogenetic analyses of influenza viruses.

Technical Appendix 2 Statistical model used for analysis of influenza viruses.
