# Supplementary material for: Swine Influenza in Sri Lanka
Source: Emerg Infect Dis. 2013 Mar;19(3):481–4. doi: 10.3201/eid1903.120945 (PMC3647653; doi:10.3201/eid1903.120945)
Supplement: Technical Appendix 1 — Methods used in phylogenetic analyses of influenza viruses. [file 12-0945-Techapp-s1.pdf]

Article DOI: http://dx.doi.org/10.3201/eid1903.120945

Swine Influenza in Sri Lanka

Technical Appendix 1

Methods

The HA sequences were aligned with other sequences from animal and human influenza A viruses available in the GenBank. Maximum-likelihood (ML) tree was estimated using the general time-reversible with invariant sites and 4 gamma distributed heterogeneous substitution rates) in PhyML version 3.0 (*1*). To evaluate the robustness of the tree topology, a set of 500 pseudo-replicates of the sequences was generated and used in bootstrap analysis. The ML tree topology was also confirmed by using Bayesian analysis in MrBayes version 3.2 (*2*).

References

1. Guindon S, Gascuel O. A simple, fast, and accurate algorithm to estimate large phylogenies by maximum likelihood. Syst Biol. 2003;52:696–704. PubMed http://dx.doi.org/10.1080/10635150390235520

2. Ronquist F, Teslenko M, van der Mark P, Ayres DL, Darling A. MrBayes 3.2: efficient Bayesian phylogenetic inference and model choice across a large model space. Syst Biol. 2012;61:539–42. PubMed http://dx.doi.org/10.1093/sysbio/sys029
