# Supplementary material for: Swine Influenza in Sri Lanka
Source: Emerg Infect Dis. 2013 Mar;19(3):481–4. doi: 10.3201/eid1903.120945 (PMC3647653; doi:10.3201/eid1903.120945)
Supplement: Technical Appendix 2 — Statistical model used for analysis of influenza viruses. [file 12-0945-Techapp-s2.pdf]

Article DOI: http://dx.doi.org/10.3201/eid1903.120945

Swine Influenza in Sri Lanka

Technical Appendix 2

Statistical Model

Monthly counts of RT-PCR testing of human samples were cumulated from January 2009 to December 2011, and then the data were smoothed by using cubic spline function assuming that each observation took place in the mid-point of the month. Subsequently, weekly human incidence was calculated by dividing weekly number of RT-PCR positive specimens from humans by the interpolated spline function. To assess possible correlation with different time-lags between swine and human epidemics, cross-correlations were estimated between estimated weekly human incidence and swine datasets. Two pieces of swine data, i.e., the proportion seropositive (seroprevalence) and the proportion of virus isolates among test samples (virus prevalence), were examined. Since the time series between human and swine may potentially have shared the trend, we also prewhitened the data. For this process, we fitted polynomial functions to the input series, got residuals from both series and computed cross-correlations from residual series.

To explore the extinction of specific virus type, we grouped the swine H1N1pdm isolates into different genetic variants based on difference in amino acids of the HA molecule. With the close homology of the isolates, a single amino acid variation was considered in grouping. When there has been no observation of a virus that belongs to a specific genetic variant for a long time, it would be useful to understand the likelihood of extinction for interpreting the absence of virus isolation in a conservative way. Because sampling a finite number of test samples during the course of an outbreak cannot prove that swine were never infected with the corresponding virus, a more useful result would be the maximum prevalence with a certain level of confidence if no positive isolates are observed among a total of *n* samples. To obtain this result, we use the following equation (1a):

$$p_{max} = 1 - \alpha^{\frac{1}{n}} \qquad (1a)$$

where p_{max} is the maximum virus prevalence given *n* samples and no positive isolates, at a confidence level of $1-\alpha$, e.g. 95% if $\alpha = 0.05$ (A1).

Appendix Results

Comparing human incidence with swine data, maximum cross-correlation between spline-interpolated weekly human incidence versus swine seroprevalence was found at 7-week lag (the cross correlation coefficient, ρ=0.545). Consistently, the maximum cross-correlation between human incidence and swine virus isolates was found at 8-week lag (ρ=0.412), indicating that the rise in swine prevalence has been seen approximately 7 to 8 weeks later than that in humans. Even after prewhitening, the maximum cross-correlations were observed at 8-week lags for both swine seroprevalence and virus isolates (ρ=0.383 and 0.348). The last isolate of virus genetic variant I, variant II and variant III took place on 14 July 2010, 8 February 2011 and 23 February 2011, respectively. Subsequently, these have not been observed even with a large number of test samples from the farms (Appendix Figure 1A), indicating that these viruses may have declined to extinction. Appendix Figure 1B shows the maximum prevalence of the corresponding genetic variants, given no observation of these viruses after the last isolation dates. The maximum prevalence lowered 0.5% in 23rd, 42nd and 46th week in 2011 for genetic variants I, II and III respectively.

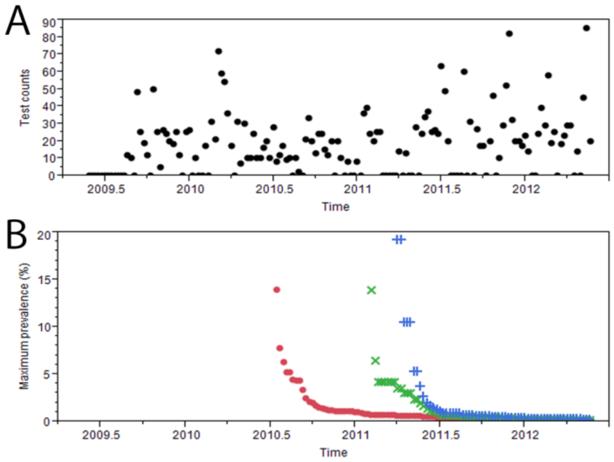

Figure. Decreasing upper bound of influenza prevalence in swine. A) Weekly number of test samples as a function of time. Despite a large number of negative test results, the negative results are informative to ensure the absence of specific viruses isolated in the past. B) Maximum influenza prevalence in swine. The estimate was calculated as the 95% upper bound of the binomial distribution give cumulative counts

of negative test results (B). The maximum prevalence for genetic variant I (filled circles), II (x marks) and III (+ mark) are shown separately.

Reference

1. Hoye BJ, Munster VJ, Nishiura H, Klaassen M, Fouchier RAM. Surveillance of wild birds for avian influenza virus. Emerg Infect Dis. 2010;16:1827–34. PubMed http://dx.doi.org/10.3201/eid1612.100589
